# Photon statistics on the extreme entanglement

**DOI:** 10.1038/srep24098

**Published:** 2016-04-07

**Authors:** Yang Zhang, Jun Zhang, Chang-shui Yu

**Affiliations:** 1 School of Physics and Optoelectronic Technology, Dalian University of Technology, Dalian 116024, China

## Abstract

The effects of photon bunching and antibunching correspond to the classical and quantum features of the electromagnetic field, respectively. No direct evidence suggests whether these effects can be potentially related to quantum entanglement. Here we design a cavity quantum electrodynamics model with two atoms trapped in to demonstrate the connections between the steady-state photon statistics and the two-atom entanglement. It is found that within the weak dissipations and to some good approximation, the local maximal two-atom entanglements perfectly correspond to not only the quantum feature of the electromagnetic field—the optimal photon antibunching, but also the classical feature—the optimal photon bunching. We also analyze the influence of strong dissipations and pure dephasing. An intuitive physical understanding is also given finally.

Nonlinear light-matter interaction is a long sought for quantum information science^1^,^2^, as well as a fascinating concept in terms of fundamental physics. The strong interactions between individual photons is a standing goal of both fundamental and technological significance^3^. Photon blockade, as a typical nonlinear quantum optical effect, which indicates the ability to control the nonlinear response of a system by the injection of single photons^4^,^5^,^6^, shows that the system ‘blocks’ the absorption of a second photon with the same energy. The typical feature is the photon antibunching which is signaled by a rise of *g*^(2)^(*τ*) with *τ* increasing from 0 to larger values while *g*^(2)^(0) < *g*^(2)^(*τ*) as discussed in detail in refs 7, 8, 9. The converse situation, *g*^(2)^(0) > *g*^(2)^(*τ*) is called photon bunching which indicates large probability of more than one photon to arrive simultaneously to the detector. It is usually considered as a purely classical behavior. As the peculiar feature of the quantum mechanical nature, photon antibunching provides a way to controlling the single photon via optical devices such as quantum optomechanical setups^10^,^11^,^12^,^13^,^14^, feed back control system^15^, superconducting circuit^16^,^17^, quantum dots^18^,^19^, Kerr-type nanostructured materials^4^, confined cavity polaritons^20^, cavity quantum electrodynamics (CQED) systems and so on^17^,^21^,^22^,^23^,^24^,^25^,^26^,^27^,^28^,^29^,^30^,^31^,^32^,^33^,^34^.

Recently, the relation between photon statistics and other quantum effects have attracted increasing interests. For example, refs 15,17 address the relation between photon blockade and optical bistability and ref. 17 also investigates the relation between photon blockade and electromagnetically induced transparency. In ref. 35 , it is found how the photon blockade is affected by the parity-time symmetry. In addition, the authors in ref. 36 find the connection between the first order correlation function and the violation of Bell inequalities. As we know, quantum entanglement is not only an intriguing quantum feature but also the important physical resource in quantum information processing^37^,^38^,^39^. Do there exist some relation between photon statistics and quantum entanglement? Or a weak question is whether one can design some particular quantum systems to create a potential relation.

In this paper, we design a particular CQED model to reveal the relations between the photon statistics and atomic entanglement. Our model includes one cavity weakly driven by a monochromatic laser field and two two-level atoms trapped in the cavity. As mentioned above, photon statistics have been widely studied in CQED systems. Even though the mechanism of photon statistics is clear, intuitively, there is no proof that photon antibunching and bunching have any direct relation with entanglement. So *our interest is mainly to find the relation between the photon statistics and the entanglement of the two atoms in a particular case instead of only illustrating the photon statistics or atomic entanglement*. Firstly, we restrict our results in the weak dissipation regime and present our main result. We find that the maximal steady-state atomic entanglements as the quantum feature just correspond to the quantum feature of the cavity field, that is, the local optimal photon antibunching. It is surprising that the local maximal steady-state atomic entanglements also perfectly correspond to the classical feature of the field, that is, the photon bunching. However, the maximal bunching point subject to a dark-state process corresponds to vanishing steady-state entanglement. Secondly, we analyze the effects of strong dissipation as well as pure dephasing on the correspondence relations. It is shown that entanglement is reduced faster than the second-order correlation function and the correspondences become worse and vanishing until the entanglement dies when the dissipations of the system are increased or the dephasing is considered. We also discuss the experimental realization of our proposal. Finally, the intuitive physical analysis and some further discussions are provided.

## Results

### The physical model

**Figure 1:**
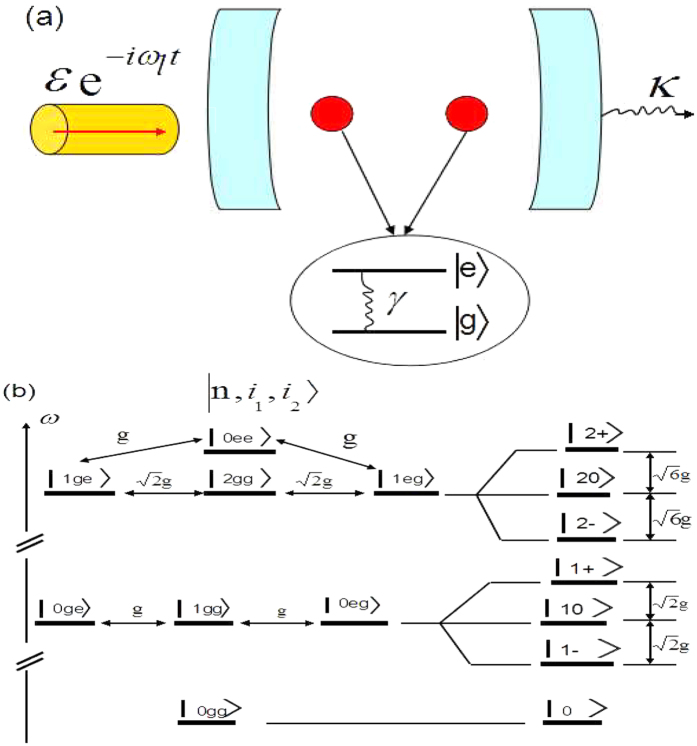
(**a**) A cavity coupled with two two-level atoms. The driving field is weakly coupled to cavity mode with Rabi frequency *ε*. *γ* and *κ* are the spontaneous emission rate of the atoms and decay rate of the cavity, respectively. (**b**) Energy levels corresponding to system’s state up to *n*_*ph*_ = 2. It indicates the relevant transition processes between states and the possible excitation pathways to state |2, *g*_1_, *g*_2_〉. The states are labeled by |*n*, *i*_1_, *i*_2_〉 with *n* denoting the photon numbers of cavity mode, and *i*_1_ and *i*_2_ representing the levels of two atoms, respectively.

As sketched in the Fig. 1(a), we study two two-level atoms coupled to the cavity with frequency *ω*_*a*_ which is weakly driven by an external optical field. The two-level atoms can be, in principle, replaced by any two-level systems such as ions, quantum dots, superconductive qubits and so on. The frequency of atomic transition from ground state |*g*〉 to excited state |*e*〉 with linewidth *γ* is denoted by *ω*_*e*_. In this configuration^40^,^41^ (we set 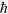 = 1 hereafter), the Hamiltonian can be given by


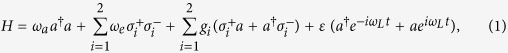


where 
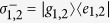
, *a*(*a*^†^) are the annihilation (creation) operators of the cavity mode and *g*_*i*_ is the coupling coefficient between the *i*th atom and the cavity mode. The driving frequency is denoted by *ω*_*L*_, and the driving strength by *ε*, respectively. In the frame rotated at the laser frequency 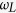, the Hamiltonian (1) becomes


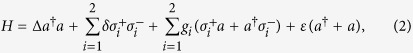


where Δ = *ω*_*a*_ − *ω*_*L*_ is the laser detuning from the cavity mode and *δ* = *ω*_*e*_ − *ω*_*L*_ is the laser detuning from the atoms.

For simplicity, here we assume that *g*_1_ = *g*_2_ = *g* and we only consider that the cavity is resonant with the atoms, i.e., *ω*_*a*_ = *ω*_*c*_ and Δ = *δ*. Since the system is driven weakly, only few photons can be excited. We can only focus on the few-photon subspace. Thus the Hamiltonian *H* without driving can be easily diagonalized (we cut off the photons into the two-photons subspace). The eigenvalues are given in the Methods and the eigenstates in the current case, distinguished by different numbers of photons, can be given as follows.


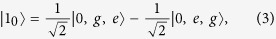



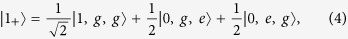



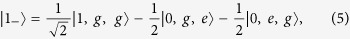



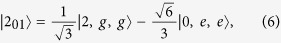



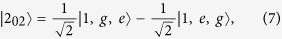



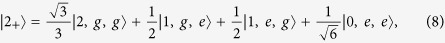



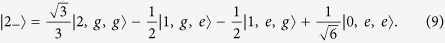


The energy levels are shown in the Fig. 1(b). The nonlinearity in the coupling between the atoms and cavity gives rise to energy level structure which can exhibit bunching and antibunching behaviors due to the splitting of the eigen-energy^42^. With this structure, we can get an intuitive picture for the different photon statistics.

### The photon statistics

As mentioned at the beginning, the different photon statistics are signaled by the equal-time (namely zero-time-delay) second-order photon-photon correlation function^43^ which reads


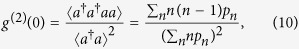


where *n* = 〈*a*^†^*a*〉 is the intra-cavity photon number of the cavity mode, *p*_*n*_ represents the probability with *n* photons. In Eq. (10) the operator is evaluated at the same time. When the second-order correlation function satisfies the inequality *g*^(2)^(0) ≤ 1, there occurs the photon antibunching, i.e., the photon blockade which means the system ‘blocks’ the absorption of a second photon with the same energy with large probability. The limit *g*^(2)^(0) → 0 means the perfect photon blockade in which two photons never occupy the cavity at the same time. On the contrary, when *g*^(2)^(0) > 1, it means that photons inside the cavity enhance the resonantly entering probability of subsequent photons^44^,^45^,^46^,^47^.

To give an intuitive picture and gain more insight into the physics, we first take an analytic (but approximate) method to calculate the second-order correlation function by employing the wave function amplitude approach. Considering the effects of the leakage of the cavity *κ*, the spontaneous emission *γ* of the atoms, we phenomenologically add the relevant damping contributions to Eq. (2). Thus the Hamiltonian can be rewritten as 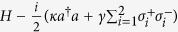. Analogous to the above statements, the photon number is up to 2. So one can assume that the state of the composite system is given by^48^,^49^


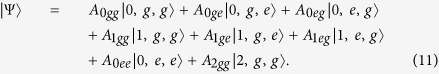


So the dynamical evolution of the state Eq. (11) subject to the damping Hamiltonian is given by


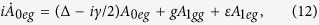



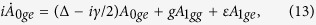



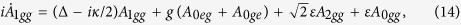



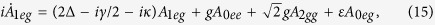



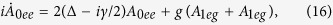



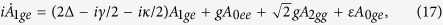



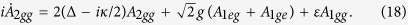


From Eq. (11), one can easily write 
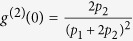
 with 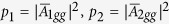. Since the weakly driving is considered, one can easily get 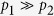, which means that *g*^(2)^(0) can be simplified as 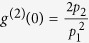. Under the stability conditions, we can easily obtain the steady-state solution of Eqs (12, 13, 14, 15, 16, 17, 18) by letting the derivatives on the left-hand-side vanish. The concrete expressions of the steady solutions are given in the Methods. Substitute the steady-state solution into *g*^2^(0), one can immediately arrive at


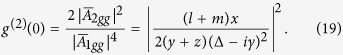


In addition, the mean photon number can also be given by


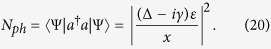


Note that here


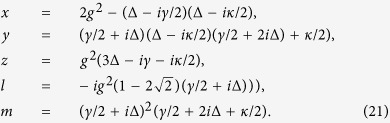


In order to show the validity of the above analytic treatment, we also employ the quantum master equation to numerically study the above results. Considering the above quantum system, the Markovian quantum master equation reads


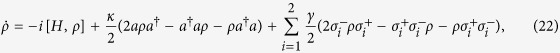


where *H* is the Hamiltonian given by Eq. (2), *ρ* is the density operator of the whole composite system, and 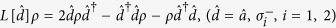 is the dissipator. In addition, we don’t consider the thermal photons for simplicity^44^. Since the steady-state solution is needed for our purpose, we will directly employ a numerical way to solving Eq. (22) for the steady state *ρ*_*s*_^50^. So the second-order correlation function can be directly obtained by 
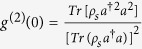
 and the mean photon number is obtained by *N*_*ph*_ = *Tr*(*ρ*_*s*_*a*^†^*a*).

**Figure 2:**
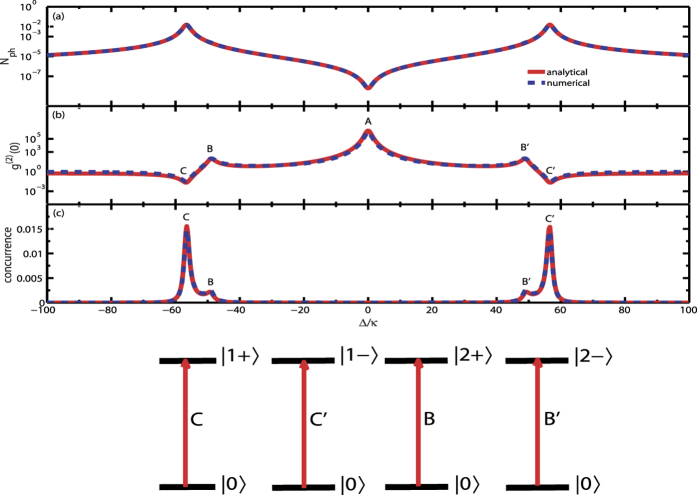
The mean photon numbers of the cavity, the equal-time second-order function *g*^(2)^(0) and the entanglement of two atoms vs the detuning Δ, respectively. The red curves are approximate and analytical solution of Eq. (20), Eq. (19) and Eq. (25). The blue curves are numerical results of the quantum master equation Eq. (22). We take *γ*/*κ* = 1, *g*/*κ* = 40, *ε*/*κ* = 0.125. At the bottom, we also show the transition between the different eigenstates corresponding to the different points.

In Fig. 2(a,b), we plot the mean cavity numbers *N*_*ph*_ and second-order correlation function *g*^(2)^(0) changing with the detuning Δ in the case of weak dissipations. One can find that the numerical results given by Eq. (22) and the analytic and approximate results given by Eqs (19) and (20) show the perfect agreement. This guarantees that all the following conclusions drawn from our analytical way is valid. Let’s focus on Fig. 2(a,b). It is shown that the points *C* and *C*′ (*g*^(2)^(0) ≪ 1) where photon statistics satisfy the sub-Poissonian distribution, correspond to the photon blockades which are the local optimal antibunching points in this system. At these two points, 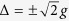 which means that the driving field is just resonant with the transition between the single-photon polariton states |1, ±〉 and the ground state |0〉. In this case, once the first photon excited the transition from |0〉 to |1, ±〉 by the coherent driving, the photon with the same energy is not resonant with any other transition (the energy does not match between any other two levels). So it seems that the first photon ‘blocks’ the absorption of a second photon. At points *B* and *B*′, one can find *g*^(2)^(0) > 1 which corresponds to the photon bunching. At these two points, 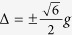 which correspond to the resonance between the driving field and the transition from the ground state |0〉 to the excited states |2, ±〉. It indicates a resonance process of double photons. At point *A*, it shows a strong photon bunching effect with *g*^(2)^(0) ≫ 1. At this point, Δ = 0 and the photon statistics satisfy the super-Poissonian distribution. This does not correspond to a resonance process. When Δ = 0, the system is coherently driven into a dark state 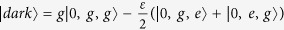. The state 
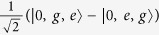
 is allowed to transit to 
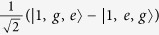
 which is strongly coupled to the state |2*gg*〉. This is similar to the electromagnetically induced transparency^51^,^52^.

### Atomic entanglement and photon statistics

Since we have calculated the state |Ψ〉 given in Eq. (11), one can easily obtain the reduced density matrix *ρ*_*AB*_ for the two atoms. Thus one can also easily calculate the corresponding entanglement. Here in order to show the two-atom entanglement, we would like to employ Wootters’ concurrence as the entanglement measure^53^ which, for the bipartite density matrix of qubits, is defined by


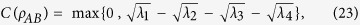


where *λ*_*i*_ is the square root of the *i*th eigenvalue of the 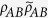 in decreasing order with 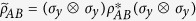. Substituting *ρ*_*AB*_ in to Eq. (23) one can easily obtain (see Methods)


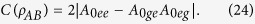


Note that in Eq. (24), we have neglected the terms with the power of *ε* more than 2. Based on the steady amplitudes derived from Eq. (6), the concurrence is analytically given by


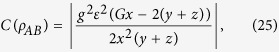


with 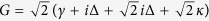.

We have plotted Eq. (25) in Fig. 2(c). The concurrence via the numerical way (by solving Eq. (22)) is also plotted in this figure. One can find that the analytic concurrence matches the numerical results very well, which guarantees the validity of our approximate and analytic results. Although the steady-state concurrence is not so large in contrast to ref. 54 which is essentially within a different mechanism (two coupled and driven cavities and relatively large driving-dissipation ratio ~10^3^), it does not affect our purpose of this paper. From Fig. 2(c), it is obvious that the concurrence has two pairs of local maximal values. Compared with Fig. 2(b), one can easily find that these two local maximal entanglement perfectly correspond to the local optimal photon antibunching and bunching. Such a correspondence can also be supported by the analytic expression given in Eq. (25), from which one can see that the extrema occur at Δ^2^ = 2*g*^2^ and 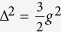 for small {*κ*, *γ*}. This is consistent with the above analysis on the photon statistics. Next we will provide give a relatively intuitive understanding of this correspondence. One should first note from Eq. (24) that only the three parameters *A*_0*ee*_, *A*_0*eg*_ and *A*_0*ge*_ play the dominant role in entanglement. So at Δ^2^ = 2*g*^2^, the driving field is tuned resonantly with the transition between |0*gg*〉 and |1, ±〉 which leads to the optimal photon blockade. In addition, the strength of such a resonant interaction is proportional to the first order of the driving field *ε*. So |1, ±〉 gets a relatively large proportion in the total state |Ψ〉. It is obvious from Eq. (24) or Eq. (25) that |Ψ〉 owns the relatively large amount of entanglement (*C* and *C*′ in Fig. 2(c)). If 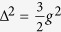, the driving field is resonant with the transition between |0*gg*〉 and |2, ±〉. Thus |2, ±〉 occupies the relatively dominant proportion in |Ψ〉. However, the interaction strength is proportional to the second order of *ε*^2^. So the entanglements at these points get the extremum (*B* and *B*′), but they are still much less than the entanglement at *C* and *C*′. We would like to point that the consistency between photon statistics and entanglement is attribute to that they can be understood in a unified and intuitive way. The resonant transitions of both single-photon process and double-photon process, as the essential physics of photon antibunching and bunching, correspond to the superposition of the ground state and an entangled state. So the maximal atomic entanglement is well consistent with photon statistics. Given the (weak) driving strength, the single-photon process happens with a much larger probability than of double-photon process, so the entanglement subject to double-photon process is small. However, it is interesting that the maximal photon bunching point at Δ = 0 does not correspond to an extremum of entanglement. The reason is attributed to the dark-state process which provides a channel (as mentioned in the part of photon statistics) to be converted to the state |2*gg*〉 as well as |0*ee*〉. Their proportions in |Ψ〉 get relatively larger. The net effect on entanglement is that |0*ee*〉 and |0*ge*〉, |0*eg*〉 reach a balance subject to Eq. (24), so the entanglement is negligibly small.

**Figure 3:**
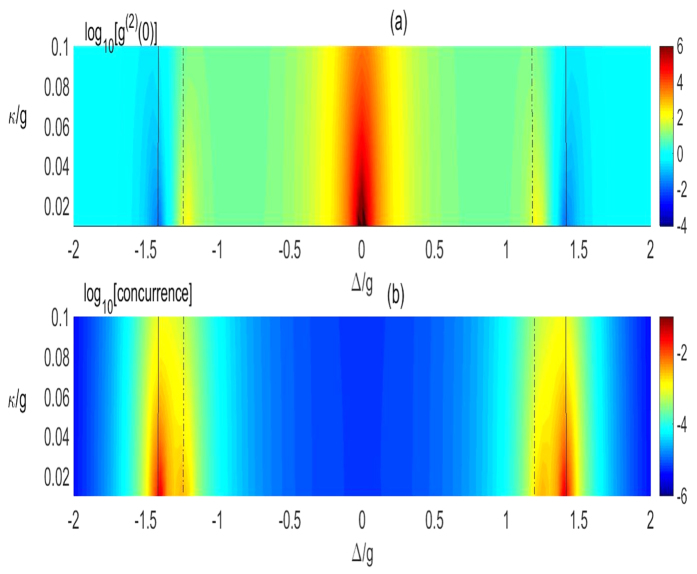
We plot logarithm of the equal-time second-order function log_10_[*g*^(2)^(0)] and the logarithm of concurrence log_10_[*concurrence*] as a function of the detuning Δ and cavity decay rate *κ*. (**a**) Shows the second-order correlation function and (**b**) corresponds to the entanglement of atoms. The locally optimal photon antibunching and bunching and the locally maximal entanglement are also illustrated by the black-solid line and black-dashed line respectively in (**a**,**b**) which corresponds to Δ^2^ = 2*g*^2^ and Δ^2^ = 3/2*g*^2^, respectively. Here, we set *γ* = *κ*, *ε*/*g* = 0.0065.

We would like to emphasize that all our presented correspondence relations hold within the weak dissipation regime. Once this condition is not satisfied, these relations will be reduced or destroyed. In order to show the influences, we first plot the concurrence and *g*^(2)^(0) via *γ* = *κ* and Δ in Fig. 3 in the vacuum environments. One can find that both the local optimal photon statistics and the concurrence extrema are reduced with *κ* increasing. Meanwhile, the correspondence relation between concurrence and *g*^2^(0) gets a little bit worse. This can be well understood from Eq. (19) and Eq. (25) from which one can see that all the relevant analysis are satisfied within the error region to the same order as *κ*^2^ (we assume *γ* = *κ* for simplicity). So we always limit our study in the region with small enough dissipations. Physically, the large deviation of the correspondence is directly attributed to the large line width of the level induced by the dissipations.

**Figure 4:**
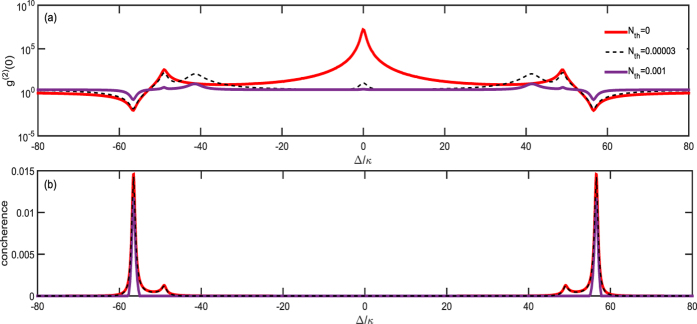
***g***^**(2)**^**(0) and the concurrence versus the detuning with different** 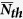. All the parameters are the same as Fig. 2. The figure shows how the correspondence is gradually destroyed by thermal environments.

Next, we will consider how thermal photons and the atomic dephasing influence our results. In fact, it can be easily predicted that our results will be destroyed greatly since quantum feature (especially the entanglement) is generally quite fragile to these environments. We consider the thermal environments by solving the following master equation 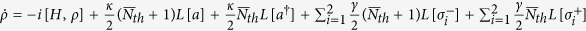 where 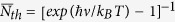 is the average photon number with *k*_*B*_ denoting the Boltzmann constant and *T* standing for the reservoir temperature. In addition, all the other parameters are defined the same as Eq. (22). The numerical results are shown in Fig. 4 where we can observe that with the increasing of 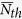, the entanglement, photon antibunching and photon bunching are all reduced at the correspondence points, but the entanglement decays very fast and even dies with large 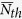. But the correspondence relations can be kept with less thermal photons until the entanglement vanishes. In addition, photon bunching can be enhanced at other places, which just shows the participation of the thermal photons. We also consider the effect of atomic dephasing procedure^55^, which is done by adding a Lindblad term 
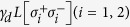
 in Eq. (22). Numerical procedure shows the completely similar results as the thermal environment. So the figures are omitted here.

### Experimental realization

Up to now, based on the schematic setup in our paper, we have theoretically studied the relation between photon blockade and the atomic entanglement and presented the physics behind this scheme. In the following, we will give a brief analysis on whether the conditions that we require are achievable in practical experiments. Based on the previous sections, we should note that the possibility to realize the proposal mainly depends on the strong coupling rate (*g*/*κ* ~ 40 and *γ*/*κ* ~ 1 for our numerical simulation). Thus, we will extensively focus on the parameters *g*, *κ* and *γ*. As mentioned above, our physical model is not restricted in the real atomic systems. Let’s consider the quantum device circuit QED system (circuit QED-consisting of microwave resonators and superconducting qubit)^56^,^57^ or quantum dot coupled with the photonic crystal cavity^58^. In circuit QED system, the strong coupling can be realized and the long coherence time of a superconducting qubit embedded in a high-quality on-chip microwave cavity^56^. The cavity-qubit coupling strengths can be realized experimentally from 2*π* × 5.8 MHz to *g*_*max*_ = 2*π* × 210 MHz and the relaxation time of the qubit can reach 7.3 *μs*^59^,^60^ which corresponds to the decay rate *γ* ~ *2π* × 0.02 MHz. The qubit transition frequencies can be chosen anywhere from about 5 GHz to 15 GHz^57^ and can be tuned by applying a magnetic flux through the qubit loop. The cavity decay rate *κ* can be as low as 2*π* × 5 KHz due to the high value of the quality factor Q with resonator frequency to be between 5 GHz and 10 GHz^57^,^61^. So the ratio used in our simulations *g*/*κ* = 40 and *γ* ~ *κ* are reasonable and easily achieved. In addition, the system can be cooled to temperatures below 20 mK^56^,^62^ (15 mK in^63^) in a dilution refrigerator. Correspondingly, the number of thermal photon 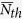 subject to the transition frequency 2*π* × 6.5 GHz for the qubit is less than 
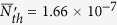
 (for 20 mK). It can even be adjusted to 
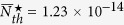
 (for 15 mK and transition frequency 2*π* × 10 GHz). From Fig. 4, one can find that the entanglement is hardly affected by 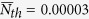, even though it is usually fragile for noise. It can be reasonably predicted that if 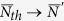, even 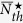, our correspondence relation will be perfectly observed in experiment. The dephasing of the qubit in one realization of this system has also been measured in refs 64,65. It shows that the pure dephasing time *T*_*d*_ can reach as long as 5.5 *μs*^65^ which translates to *γ*_*d*_ = 2*π* × 0.03 MHz ≈ 1.5 × 10^−4^*g*_*max*_. We can loosely choose *κ* and *γ* such that 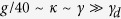 is achieved, so the effect of dephasing can be safely omitted here^66^. One can find that the pure dephasing is direclty omitted in ref. 63 by adjusting the experimental parameters. Based on the above analysis, one can easily find that all the conditions required for the demonstration of the correspondence relation are realizable within the current experimental technology.

## Discussion

To sum up, we have analyzed the physical mechanisms of photon statistics and entanglement in detail. We find that the local maximal entanglement always correspond to the local optimal photon bunching and antibunching points. In other words, the local extremum of photon statistics subject to the resonance processes are in good agreement with the local maximal entanglement. However, the maximal photon bunching point corresponds to the almost vanishing entanglement due to the dark-state process. One could think that the correspondence between atomic entanglement and photon antibunching could be easily understood since both of them are the quantum feature, whereas it could be strange that the quantum feature (atomic entanglement) corresponded to a classical effect (photon bunching). We also consider how the correspondence is affected by thermal noises and pure dephasing.

In addition, we would like to provide a qualitative physical interpretation again. In CQED model, the photon antibunching essentially corresponds to resonant transition between the ground state and the single-excitation eigen-modes and bunching corresponds to the transition between the ground state and the two-excitation eigen-modes. Once such transitions happen, the trapped double atoms have 50% probability to only absorb one photon to form a maximally-entangled-state component in the corresponding eigen-mode. This is the key matching mechanism. So photon statistics corresponding to such transition procedures are consistent to the extremum entanglement. But the double-excitation procedures happen with relatively little probability due to the weak driving, so the entanglement is much smaller. All the above analysis are obviously limited under the condition that the incoming photon (energy) can be well kept and no extra photons disturb this matching mechanism. This just means the weak dissipation. On the contrary, the strong decays (*κ* and *γ*), the large thermal photon number as well as the dephasing reduce and even break the matching relation, so the correspondence gets worse. The dark-state process is another path which reaches the photon bunching around the mentioned matching mechanism, so there is no entanglement at this point. Therefore, we emphasize that the correspondence should be taken into account within weak dissipations. The proposal is within reach by current technologies, especially in the state-of-the-art circuit QED system.

Finally, we want to say that there are other relevant questions deserving us forthcoming efforts. For example, is there other mechanism leading to such a correspondence, or can we find other models with stronger correspondence? Can we effectively use this relation to control photon statistics by entanglement, or on the contrary, to control entanglement by photon statistics?

## Methods

### Eigenvalues of Hamiltonian

The Hamiltonian without driving in Eq. (2) can be easily diagonalized in few-photon subspace. For integrity, here we would like to provide the concrete expressions of the eigenvalues of the Hamiltonian. Note that the driving frequency is retained and we cut off the photon number up to 2.


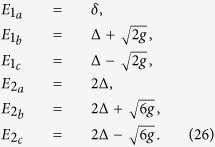


### The steady-state solution of Eqs (12–18)

In order to obtain the steady-state solution of Eqs (12, 13, 14, 15, 16, 17, 18), we set the time derivatives to be zero and solve the equations within the weak driving limit. We assume 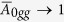, and drop the terms of the power of *ε* more than 2. The solutions are given as follows.


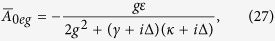



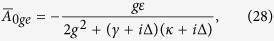



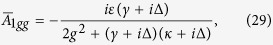



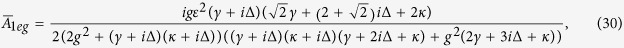



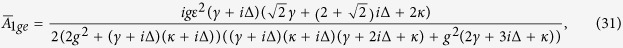



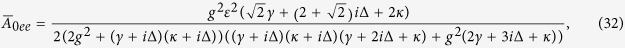



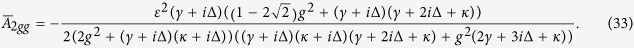


### Concurrence of the two atoms

Here we give a detailed derivation of the concurrence given in Eq. (24). Since we have obtained the steady-state solution of Eqs (12, 13, 14, 15, 16, 17, 18), one can find that the photon number and the excitation number in the subscript of *A*_*ijk*_ signal the power of *ε* in *A*_*ijk*_. From the state |Ψ〉, one can find that the reduced density matrix of the two atoms can be given by


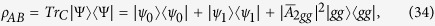


where


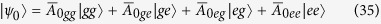



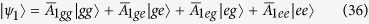


and the subscript *C* means trace over cavity field. In order to calculate the concurrence defined by Eq. (23), we need to calculate the matrix ρSρ*S with 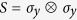. Thus one can have


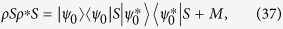


with


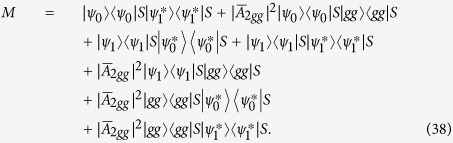


Since 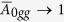, 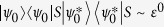 and 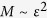. Thus *M* can be regarded as the perturbation. To proceed, we can find that the eigenvalue and the left and right eigenvectors of the matrix 
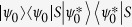
 are 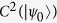, 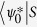 and 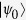, respectively. So the ‘first-order’ correction of the eigenvalue (*C*^2^(|*ψ*_0_〉)) can be given by 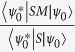. Note that 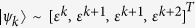, *k* = 0, 1 and the matrix *S* is anti-diagonal. One can easily find that 
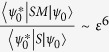
. Thus to a good approximation, the eigenvalue of ρSρ*S is well determined by 
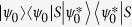
 which means the concurrence reads (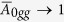)


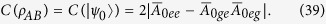


## Additional Information

**How to cite this article**: Zhang, Y. *et al*. Photon statistics on the extreme entanglement. *Sci. Rep*. **6**, 24098; doi: 10.1038/srep24098 (2016).
